# Language matters: the case of “febrile neutropenia”

**DOI:** 10.1007/s15010-026-02759-1

**Published:** 2026-03-04

**Authors:** Enrico Schalk

**Affiliations:** Department of Hematology, Oncology and Cell Therapy, Medical Faculty, Otto von Guericke University Magdeburg, Leipziger Str. 44, 39120 Magdeburg, Germany

**Keywords:** Neutropenia, Neutropenic, Febrile, Fever, Infection

Dear Editor,

Language reflects our thinking, and our thinking steers our actions [1]. In medicine, only the correct terms and diagnoses lead to correct therapies. Fätkenheuer discussed that in an example of “septic arthritis”: Whether or not the patient has a sepsis makes a big difference in the management of infectious (“septic”) arthritis (e.g., [i] *Staphylococcus aureus* has grown from pus from knee puncture; or [ii] the purulent material aspirated during arthroscopy remained sterile) [1]. Incorrect, ambiguous terms—once introduced—persist in medical literature, as shown for “septic arthritis” with about 9,000 publications from 2016, the year the “Third International Consensus Definitions for Sepsis and Septic Shock” were released [1].

Insisting on clear and precise language is not only an academic exercise [1], but also important for fundamental considerations, where it is worthwhile to ask *The Thinker* [2]. In medicine: Who or what are we treating—patients, diagnoses, or lab results? Language matters: the case of “febrile neutropenia” (Table 1).

**Table 1:** Condition and terms

*Condition*
(1.) Absolute neutrophil count < 500/µL (agranulocytosis)
- or < 1000/μL with predicted decline to < 500/μL within next 2 days
(2.) Fever ≥ 38.3 °C (101°F) once
- or ≥ 38.0 °C (100.4°F) lasting for at least 1 h or measured twice within 12 h
(3.) Infectious cause
*Most common term for the condition*
(1.) Febrile neutropenia
*Other terms for the condition*
(1.) Neutropenic fever
(2.) Fever and neutropenia
(3.) Fever in neutropenia
(4.) Febrile episode in neutropenia
(5.) Fever during neutropenia

First, “neutropenia” is a laboratory parameter [3], sometimes without but often with medical relevance [4, 5]; decades ago, identified by Bodey, an absolute neutrophil count of 500/µL as the critical threshold for infection risk [5]. Second, “febrile” is an adjective—describing or defining a noun/person, pronoun or state—, relating to, caused by, or characterized by a fever. However, *The Thinker’s* reflection on this:

(i) neutropenia relating to a fever—this is possible.

(ii) neutropenia caused by a fever—that’s is not possible directly.

(iii) neutropenia characterized by a fever—what is that supposed to be?

Are we treating “febrile neutropenia”? Probably not, but rather a patient with a fever who is neutropenic. Fever is a symptom of infection that has been around for millennia. And this is what we treat: fever, the patient with infection and neutropenia (i.e., neutropenic [infectious] fever; Table 1). Even though “febrile neutropenia” is less clear in terms of language and content than “neutropenic fever”, “febrile neutropenia” is found fivefold more often in the literature [6].

On the other hand, nobody would say “febrile leukocytosis” if one meant a patient with fever and elevated white blood cell count due to bacterial infection, or “febrile elevated transaminases” in the case of acute viral hepatitis.

Everyone can imagine a sick person with a fever, everyone knows about fever, but how does one imagine neutropenia? “Neutropenic fever” puts the patient, the person, more at the center, thus ensuring a more person-first language [7] – with more implications for action.

## Data Availability

Not applicable.
